# Frequency drift during intensive SSFP scanning: implications and solution for 3T neonatal CMR

**DOI:** 10.1186/1532-429X-15-S1-P5

**Published:** 2013-01-30

**Authors:** Anthony N Price, Shaihan J Malik, Kathryn Broadhouse, Anna Finnemore, Giuliana Durighel, David J Cox, AD Edwards, Alan Groves, Joseph V Hajnal

**Affiliations:** 1 Division of Imaging Sciences and Biomedical Engineering, King's College London, London, UK; 2 Robert Steiner MRI Unit, Imaging Sciences Department, Imperial College London, London, UK

## Background

Balanced-SSFP is widely used because of its inherent high-contrast and high-SNR efficiency, and therefore is an obvious choice for neonatal cardiac applications. Typically, multiple averages are needed due to the high-spatial and temporal resolutions required, especially for the smallest preterm infants. Therefore, prolonged intensive scans are required placing high demands on scanner hardware. Consequently, one of the two critical prerequisites for successful SSFP - sufficient B0 shimming and stable scanner frequency - is often not met using standard protocols.

## Methods

Frequency drifts were assessed, alongside a method of active correction, at 3T on phantoms and in vivo. A prolonged SSFP protocol, optimised for neonates, consisted of a multi-2D cine stack, retrospectively-gated to 20 cardiac phases, 1x1 mm resolution in-plane (FOV=10x10 cm), 4 mm slices (10 with ~1 mm overlap) and 8 averages (TA~10 mins, FA/TE/TR=35/1.9/3.8 ms). In phantoms an additional shim offset was applied to introduce band artifacts and allow visualisation of frequency drift.

Additional tests were performed using 3D SSFP acquired at 1x1x1 mm (FOV=10x10x3 cm), 20 phases, 1 average, TA~5 mins and repeated several times to assess whether drifts also occur in 3D protocols.

An active frequency stabilisation (FS) method was adapted and implemented into the multi-2D sequence. FS was achieved by monitoring phase differences between two gradient echo readouts applied between every slice increment, and compared to baseline. The FS stack protocol was performed on 18 infants (CGA: 27+5 to 40+3 weeks, weight: 610 to 3120 g). B0 mapping and image based shimming was used to ensure adequate B0 uniformity over the neonatal heart.

## Results

**Figure 1:**
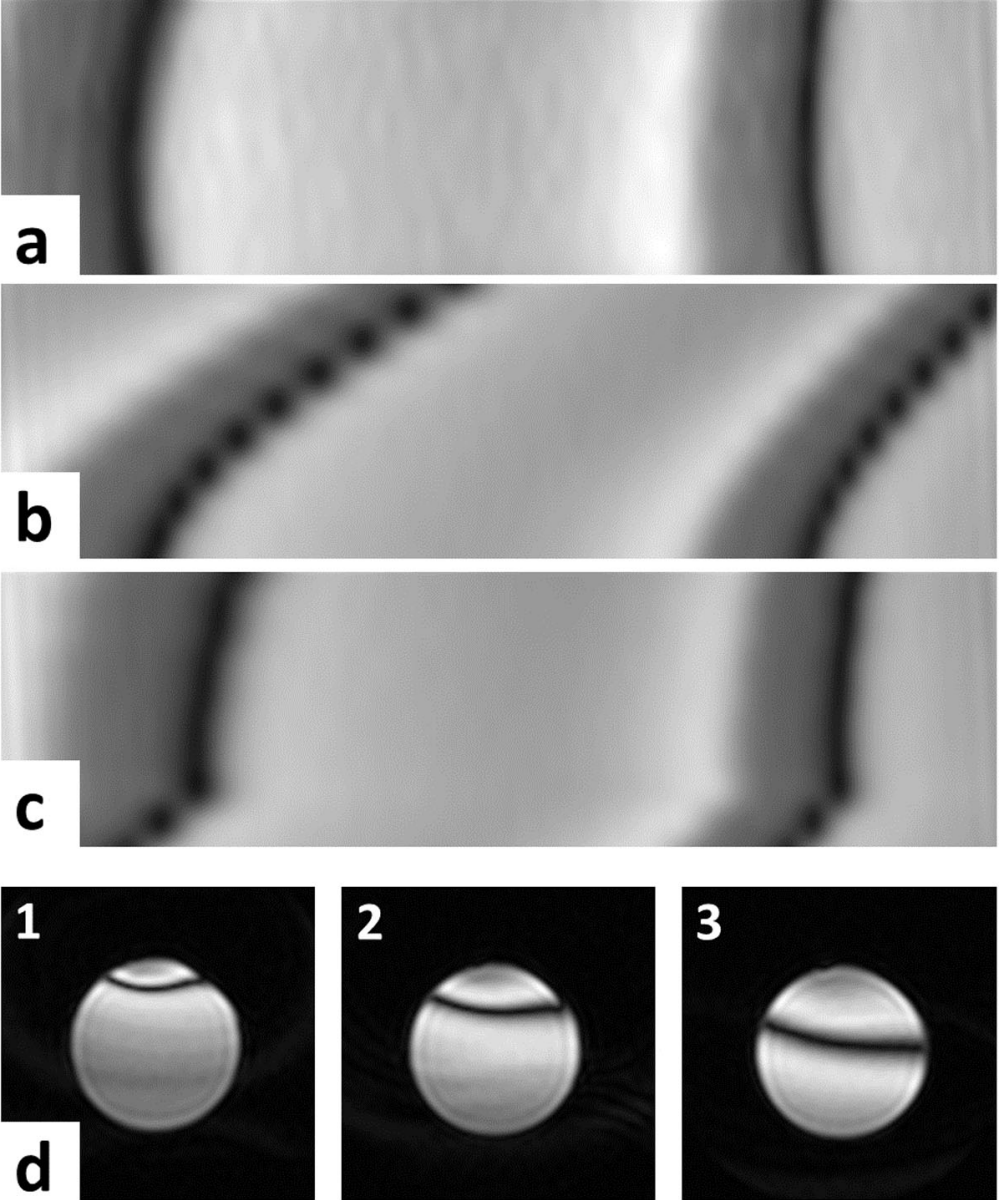
Frequency drift test in phantoms during prolonged SSFP: reformatted views (phase-slice direction) of a multi-2D stack acquisition in (a) fast reference scan - no drift, (b) neonatal optimised protocol and (c) added frequency stabilisation. Single slice view from (d) a 3D cine SSFP protocol also show a progressive frequency drift through repeated scan acquisitions 1-3.

**Figure 2:**
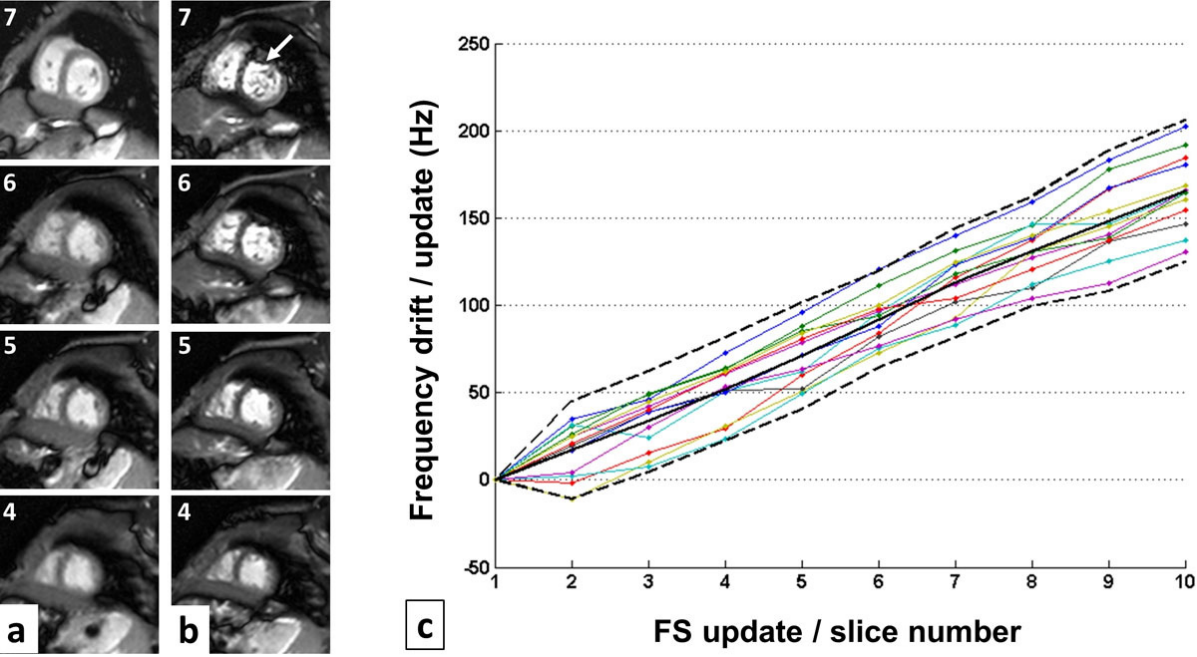
Neonatal cine SSFP stack acquisition (showing slices 4-7 from 10, apex-base order) (a) with and (b) without the proposed frequency stabilisation. The accumulated frequency drift updates performed during stack acquisitions in 13 infants is shown (c), the continuous black line represents the mean and the dashed line the 95% confidence intervals for each FS update/slice increment.

The phase-slice reformats in Figure 1 reveal progressive drift in bands, and thus frequency, up through the prolonged SSFP stack protocol, compared to the reference scan. Repeating this with frequency stabilisation shows bands following a similar pattern to the reference from slice 3 onwards - suggesting stabilised frequency. Drifts were also observed using the 3D protocol between each repetition of the scan (TA~5mins). The *in vivo* example in Figure 2 reveals frequency drift severely degrading images by slice 7, but rectified using frequency stabilisation. Artifact-free cine stacks were acquired in 16 infants, the frequency drift observed over 13 cases (Figure 2c) reveals an average drift of 165 Hz, over the 10-minute protocol.

## Conclusions

Significant drifts were found on a 3T system hampering the use of prolonged SSFP for neonatal CMR. Active frequency stabilising was achieved, vastly improving image quality and allowing for adequate volume segmentation. Frequency drifts may also affect adult CMR protocols requiring prolonged SSFP, such as free-breathing whole-heart cine or MRA.

## Funding

MRC Clinician Scientist Fellowship

